# Musical training as an alternative and effective method for neuro-education and neuro-rehabilitation

**DOI:** 10.3389/fpsyg.2015.00475

**Published:** 2015-04-28

**Authors:** Clément François, Jennifer Grau-Sánchez, Esther Duarte, Antoni Rodriguez-Fornells

**Affiliations:** ^1^Department of Basic Psychology, University of Barcelona, Barcelona, Spain; ^2^Cognition and Brain Plasticity Group, Bellvitge Biomedical Research Institute, Barcelona, Spain; ^3^Department of Physical Medicine and Rehabilitation, Parc de Salut Mar, Hospitals del Mar i de l’Esperança, Barcelona, Spain; ^4^Catalan Institution for Research and Advanced Studies, Barcelona, Spain

**Keywords:** neuro-rehabilitation, neuro-education, music training, music therapy, stroke rehabilitation, language development disorders

## Abstract

In the last decade, important advances in the field of cognitive science, psychology, and neuroscience have largely contributed to improve our knowledge on brain functioning. More recently, a line of research has been developed that aims at using musical training and practice as alternative tools for boosting specific perceptual, motor, cognitive, and emotional skills both in healthy population and in neurologic patients. These findings are of great hope for a better treatment of language-based learning disorders or motor impairment in chronic non-communicative diseases. In the first part of this review, we highlight several studies showing that learning to play a musical instrument can induce substantial neuroplastic changes in cortical and subcortical regions of motor, auditory and speech processing networks in healthy population. In a second part, we provide an overview of the evidence showing that musical training can be an alternative, low-cost and effective method for the treatment of language-based learning impaired populations. We then report results of the few studies showing that training with musical instruments can have positive effects on motor, emotional, and cognitive deficits observed in patients with non-communicable diseases such as stroke or Parkinson Disease. Despite inherent differences between musical training in educational and rehabilitation contexts, these results favor the idea that the structural, multimodal, and emotional properties of musical training can play an important role in developing new, creative and cost-effective intervention programs for education and rehabilitation in the next future.

## Introduction

Recently, the Organisation for Economic Co-operation and Development published the “2012 PISA report” providing strong evidence of the dramatic drop in the scholar level of 15-year-old French pupils over the past 10 years (OECD, 2014). Compared to data collected in 2003, 15 years old French children exhibited a drop of 15 points (from 511 to 495) in mathematics and the number of pupils in difficulty increased dramatically. In addition, learning disorders are very frequently diagnosed during childhood with the prevalence of developmental dyslexia being of 7–10% of the general population (Démonet et al., 2004; Collective expertise INSERM, 2007). These data point toward the urge of building and testing efficient learning tools to optimize the learning trajectories of typically developing young pupils and even more importantly to remediate specific disabilities found in children with language-based learning impairments. In addition to the burden in the young population, the demographic changes in life expectancy will lead to a significant increase of the population aged over 65 years old in Europe. This population is at high risk of suffering from neurologic age-related diseases (Salomon et al., 2012). For instance, the incidence of stroke is expected to grow by 36% from 2000 to 2025 (Truelsen et al., 2006). The last study of the World Health Organization Global Burden of Disease project is eloquent in showing that stroke remains the second cause of worldwide mortality (Lozano et al., 2012). Although advances in the acute medical management of stroke patients have reduced mortality in high-income countries (Feigin et al., 2014), stroke is still a major cause of disability-adjusted life-years (Murray et al., 2012). Beyond cardiovascular diseases, the rapid aging in Europe will also increase other non-communicable disorders such as neurodegenerative diseases. For instance, Parkinson’s disease affects 2.4 per 100 inhabitants of more than 65 years and its prevalence is expected to double by year 2030 (Dorsey et al., 2007). In this context, the need to develop innovative and effective rehabilitation evidence-based techniques is a challenge for the next years in the field of neuro-rehabilitation.

During the last decade, the neuroscientific community has developed a line of research on music perception and on the musician’s brain. Results obtained have largely contributed to increase our knowledge on the brain functioning in general and have allowed delineating the positive impact of playing a musical instrument on brain plasticity. In the first part of the review, we report evidence from cross-sectional and longitudinal studies showing that learning to play a musical instrument can induce substantial neuro-plastic changes in cortical and subcortical regions of motor, auditory and speech processing networks. The second part focuses on music to language transfer effect and on the necessary conditions for enhancing language processing in healthy participants. We follow by reporting an overview of the evidence showing that musical training can be an alternative, low-cost, and effective method for the remediation of language-based learning impaired populations. We then focus on neuro-rehabilitation by presenting the results of studies showing that music interventions can enhance motor recovery and neuroplasticity after stroke and can ameliorate motor deficits observed in Parkinson disease. Finally we discuss some of the important similarities and differences between musical training for neuro-education and for rehabilitation purposes.

## Musical Practice Fosters Neuroplasticity

In the last decade, the neuroscientific community has concentrated a great amount of effort to explore the positive impact of playing a musical instrument on the brain. Those efforts have provided converging evidence that the musician’s brain is an excellent model of neuroplasticity, specifically in the sensory-motor system (Münte et al., 2002; Zatorre, 2013). Indeed, playing in a symphony orchestra requires a large amount of practice: 20-year-old orchestra musicians typically spend more than 10,000 h of musical practice (Krampe and Ericsson, 1996). During all those hours, the musician will develop and ultimately master many different competences involving sensory-motor, mnesic, cognitive control, and attentional processes. As a consequence of the repetition of this complex activity, the underlying neural substrates will be eventually modified due to functional and structural neuroplastic mechanisms (see for a recent review, Kolb and Muhammad, 2014).

### Neuroplastic Changes in the Sensory-Motor Network

The neuroplastic changes induced by specific training can be studied using both cross-sectional and longitudinal approaches. While longitudinal studies generally use a test-training-retest procedure with naïve participants before training, cross-sectional studies compare a group of experts to a group of laymen participants. In the case of cross-sectional studies in which eventual pre-existing inter-individual differences might account for the differences observed between the groups (Zatorre, 2013) so that causality between music training and the observed effects cannot be demonstrated. By contrast, longitudinal studies with pseudo-random assignment of the participants to a training group and a non-training group allow determining that musical training is the cause of the differences (Schellenberg, 2004). Using a cross-sectional approach, Bangert and Schlaug (2006) conducted a study comparing the anatomical structure of the primary motor cortices in professional musicians and non-musicians. Using magnetic resonance imaging (MRI) these authors compared a group of non-musicians to pianists and violinists. While pianists showed similar structural modifications over both hemispheres, violinists showed a modification only over the right hemisphere. Indeed, playing the piano or the violin involves different type of bimanual control: pianists require fast and accurate finger movements of both hands whereas violinists use both hands asymmetrically favoring fine motor control of left hand fingers and gross motor control of the right hand thus leading to such a structural asymmetry. It is interesting to note that these studies provided evidence on how practicing particular cognitive and motor induce structural plastic brain alterations and improved level of performance for instance in motor areas (Schlaug et al., 1995a; Schlaug, 2001; Schmithorst and Wilke, 2002; Gaser and Schlaug, 2003; Hutchinson et al., 2003; see also Elbert et al., 1995; Draganski et al., 2004; Bengtsson et al., 2005).

Interestingly, the level of musical practice seems to be positively correlated with the increase in gray matter (GM) over motor regions (Gaser and Schlaug, 2003). Nonetheless, a recent study using particularly well controlled and highly selected pianists challenged the results mentioned above by showing a more complex pattern with decreasing GM density in peri-rolandic and striatal areas together with increasing GM density over areas involved in higher order processing such as the right fusiform gyrus, the right mid orbital gyrus, and the left inferior frontal gyrus (James et al., 2014). Increases in GM volume in the putamen have also been associated with timing variability and irregularity of scale playing in professional pianists (Granert et al., 2011). In this study, it was also observed that patients with musical dystonia presented more volume of GM in the right middle putamen. These surprising results may relate to the age of onset of musical practice and to excessive training, a potentially important factor for influencing plastic mechanisms and neural efficiency (Amunts et al., 1997). For instance, the age of onset of musical training is correlated with the level of motor performance in a rhythm synchronization task (Bailey et al., 2014) and may be decisive for the non-linear dynamic of structural brain changes induced by musical practice (Steele et al., 2013; Groussard et al., 2014).

Musical practice can also modify the strength of the connections between distant areas via white matter modifications. Compared to non-musicians, professional pianists exhibit a larger anterior portion of the Corpus Callosum, the main white matter fiber bundle connecting the two hemispheres (Schlaug et al., 1995b). Using diffusion tractography imaging method (DTI), it has been recently reported that musicians exhibit stronger white matter connectivity in the left and right supplementary motor areas (Li et al., 2014), in the corticospinal tract (Imfeld et al., 2009) and importantly, between auditory and motor areas (Oechslin et al., 2010; Halwani et al., 2011). The strong coupling of motor and auditory networks has been confirmed by further functional imaging studies showing activation of motor areas during the listening to musical rhythms in musicians (Haueisen and Knösche, 2001; Grahn and Brett, 2007; Bengtsson et al., 2009; Grahn and Rowe, 2009). Nevertheless, non-musicians also show such audio–motor co-activation (Baumann et al., 2007).

Moreover, short-term training programs also induce functional plastic changes. When beginners learn to play simple melodies (Bangert and Altnemüller, 2003). For instance, Lahav et al. (2007) trained non-musicians to play melodies by ear during five consecutive days and found activations in premotor regions when participants passively listened to the trained melodies (see also Meister et al., 2005). By contrast, Chen et al. (2012) observed a reduction of activation in both dorsal and ventral premotor cortices during musical training of naïve participants. This pattern of reduced activation over motor areas in musicians may reflect functional efficiency changes induced by musical training (Jäncke et al., 2000). In this context, Pascual-Leone et al. (1995) were the first in observing functional reorganization during piano learning using transcranial magnetic stimulation (TSM). After 4 weeks of training, participants showed a reduction of the motor map while showed an increase during the first week. This reorganization process over cortical motor maps highlight three important aspects. Firstly, rapid functional changes can occur after a rather short period of training. Secondly, the plastic changes induced by short-term training disappear when the training ends (see for example also Draganski et al., 2004). Thirdly, long-term training can lead to more efficient reduced patterns of activation. Aside from motor regions, plastic changes have also been reported at the level of the somatosensory cortices: musicians are more sensitive to tactile stimulations of the fingers than non-musicians, suggesting that musical practice can modify the size of the somatosensory receptive fields (Elbert et al., 1995).

Playing a musical instrument requires a clear motor component for a good performance. Nonetheless, the auditory dimension is also crucial in order to generate error feedback that might correct or adjust movements in case of errors (Maidhof, 2013; Maidhof et al., 2013; Pfordresher and Beasley, 2014) and to accurately perceive the pertinent acoustical parameters of the auditory input. Therefore, music induced plastic modifications over auditory areas might also be expected to occur.

### Neuroplastic Changes in the Auditory Pathway

The positive effects of musical practice on auditory processing have been evidenced by several studies showing lower discrimination thresholds for frequency, duration, silences, or time intervals in experts than in laymen (Jones et al., 1995; Kishon-Rabin et al., 2001; Micheyl et al., 2006; Rammsayer and Altenmüller, 2006; Mishra et al., 2014). In terms of structural plasticity, as in the case of the motor system, musical practice induces structural changes in the cortical auditory network: GM changes have been observed in both longitudinal and cross-sectional studies with musicians exhibiting enlarged AC compared to non-musicians (Schlaug et al., 1995a; Keenan et al., 2001; Schneider et al., 2002, 2005; Bermudez and Zatorre, 2005; Hyde et al., 2009). Moreover, compared to non-musicians, musicians also show more developed superior longitudinal fasciculus and arcuate fasciculus, the fiber bundles connecting the AC to Broca’s area (Oechslin et al., 2010; Wan and Schlaug, 2010; Halwani et al., 2011).

In line with these findings, functional changes have been reported in musicians at almost every single step of the auditory pathways: from the cochlea to the inferior colliculus (IC) and finally to the auditory cortices (AC). Musicians show functional changes already at the very peripheral level with enhanced activity of the Medial olivocochlear complex, responsible for controlling the cochlear micromechanics (Perrot et al., 1999; see for a review Perrot and Collet, 2014). Since almost 10 years, the systematic work of Nina Kraus and her group have provided exciting evidence that musical practice also induces substantial neuroplastic changes over subcortical structures. The IC, a subcortical structure in the brainstem receiving both bottom-up inputs from the cochlea and top-down inputs via the corticofugal pathway (Kraus and Chandrasekaran, 2010), encodes specific characteristics of the auditory input (Russo et al., 2004). For instance, very brief acoustic events such as stop consonants (“b,” “p,” “g,” “d,” “t”) are reflected by the transient response of the auditory brainstem responses (ABRs) whereas sustained acoustic events such as vowels are reflected by the frequency following response (FFR). Compared to non-musicians, adult musicians show more robust transient response and FFR to both musical and speech sounds (Musacchia et al., 2007; Parbery-Clark et al., 2012) suggesting that at the level of the brainstem, the representations of the sounds are more elaborated and more accurate in musicians than in non-musicians. The enhancement of ABRs is already visible at age three suggesting that very few years of musical training might be sufficient to elicit such consistent plastic changes in the IC (Strait et al., 2013). Moreover, the benefit of musical training received during childhood appears to remain during adulthood as revealed by a correlation between ABR amplitude and how recently participants quitted the training (Skoe and Kraus, 2012; see also Strait and Kraus, 2011).

Finally, musical practice fosters functional brain plasticity in cortical areas: compared to non-musicians, adult and child musicians show enhanced response of the AC as reflected by larger N1/P2 amplitude to complex sounds (Shahin et al., 2003, 2004; Trainor et al., 2003). Moreover, this enhanced N1/P2 is particularly visible when the stimulus presented is the instrument played by the participants (Pantev et al., 1998, 2001). Again, these results suggest that musicians have a more elaborated representation of the auditory input and better encode fine-grained features of sounds than non-musically trained individuals.

Interestingly, musical practice also increases the neural sensitivity to the statistical regularities found in the auditory input (François and Schön, 2014). For instance, a deviant sound rarely occurring within a sequence of repeated standard sounds elicit a specific event-related potential (ERP) component, the mismatch negativity (MMN), reflecting the pre-attentive detection of an auditory change (Näätänen et al., 2005; Grimm and Escera, 2011). Adult and child musicians often show larger and/or earlier MMNs to changes in several features of the sounds such as frequency, duration, intensity, or spatial localization than non-musicians (Tervaniemi et al., 2001, 2005; Van Zuijen et al., 2005; Vuust et al., 2005, 2011; Brattico et al., 2009; Marie et al., 2011; Putkinen et al., 2014). Additional evidence shows that musicians exhibit enhanced MMN to change in longer sequences of structured sounds (Tervaniemi et al., 2001; Trainor et al., 2002; Fujioka et al., 2004; Habermeyer et al., 2009).

## Neuro-Education: Music Training as an Alternative Tool to Promote Literacy Skills

After having delineated the positive effects of playing music on brain plasticity in the auditory and motor networks, we now turn to the topic of neuro-education We aim to give an overview of evidence showing that language-based learning impaired children often show deficits in the specific processes that are boosted by musical practice. We then present the hypothesis of music to language transfer effect, which allow grounding the subsequent evidence from both cross-sectional and longitudinal studies showing the beneficial effects of musical practice on speech processing in typically developing children. We finally summarize the few studies testing music-training programs in language-based learning impaired populations.

### Speech Processing in Children with Language Impairment

Children with language based learning impairments such as developmental dyslexia present a specific deficit in reading despite conventional instruction, socio-cultural level, normal intelligence and the absence of sensory deficits (Lyon et al., 2003; Vellutino et al., 2004; Collective expertise INSERM, 2007). Phonological awareness is crucial for normal language development (Ramus, 2003; Serniclaes et al., 2004) and relies on the ability to categorize speech sounds on the basis of extremely short timing differences. The voice onset time (VOT) is an acoustic parameter largely used to study phonological awareness as it allows differentiating the sound “ba” from the sound “pa,” a task in which dyslexic children have difficulties (Serniclaes et al., 2004). In line with these findings, Chobert et al. (2012) were able to demonstrate that children with dyslexia are impaired in the pre-attentive processing of VOT and duration in syllables based on MMN data. Children with language learning difficulties have also difficulties in extracting speech sounds when presented in a background noise (Ziegler et al., 2005, 2009) and show degraded neural responses to speech in noise stimuli (Warrier et al., 2004; Wible et al., 2004; Anderson et al., 2010).

These children might present a general deficit in the processing of timing information (Goswami et al., 2011). This timing hypothesis would explain why they exhibit impaired phonological processing (Ziegler and Goswami, 2005) and impaired general rhythmic processing (Thomson and Goswami, 2008; Corriveau and Goswami, 2009). Huss et al. (2011) showed that children with dyslexia present a deficit in the perception of rise time, an acoustic parameter important to extract the periodic and eventually metrical structures of speech (Cummins and Port, 1998; Quené and Port, 2005; Goswami, 2010; Huss et al., 2011). Growing evidence converge on the idea that rhythmic skills are crucial for the development of literacy skills in typically developing children (Tierney and Kraus, 2013; Woodruff Carr et al., 2014) and that children with dyslexia are impaired at tapping to a rhythm, and in perceiving tempo (Thomson and Goswami, 2008; Corriveau and Goswami, 2009). These results also confirm findings showing a link between literacy skills, phonological abilities, and musical aptitudes in typically developing child and in adult participants (Anvari et al., 2002; Slevc and Miyake, 2006; Tierney and Kraus, 2013). Following this line, a recent study with 48 children with dyslexia shows that temporal auditory processing strongly predicts phonological processing and reading abilities (Flaugnacco et al., 2014).

### Music to Language Transfer of Competences: Why and How Music Can Transfer to Language

Transfer of skills generally occurs when a specific skill acquired in one specific domain influences processes in another supposedly unrelated domain. Several observations have lead to the hypothesis that musical practice could transfer to language and more specifically to speech processing (Kraus and Chandrasekaran, 2010; Besson et al., 2011; Patel, 2011, 2014). Firstly, as presented above, there is a whole body of literature showing enhanced auditory processing in musicians. Secondly, music and speech share similarities: both involve the processing of similar acoustic cues (such as pitch, intensity, timbre, and duration) and both involve maintaining sequences of sounds that are unfolding in time in a structured manner. Thirdly, music and speech processing show a clear overlap in their cortical and subcortical neural substrates (Koelsch et al., 2005; Vigneau et al., 2006; Schön et al., 2010), suggesting shared neural resources (Patel, 2011, 2014).

### Music to Language Transfer Effects, Evidence in Healthy Children

In the specific context of education, it is now clearly demonstrated that noisy environments in classroom settings with higher than normal ranges level of noise negatively impacts pupils’ performance (Knecht et al., 2002; Shield and Dockrell, 2008). Indeed, the level of performance in a syllable discrimination task dramatically drops as the level of noise increases (Neuman et al., 2010). The amplitude and latency of ABRs are also clearly reduced in the presence of acoustic noise (Burkard and Sims, 2002; Russo et al., 2004). Children with language based learning impairment show impaired speech in noise perception and altered neural responses to speech sounds presented in a background noise. Musical practice seems to be a good tool to prevent this deleterious effect of noise as adult musicians exhibit more preserved ABRs in noise than non-musicians (Parbery-Clark et al., 2009a,b; Bidelman and Krishnan, 2010; Strait et al., 2012). Furthermore, a recent longitudinal study has revealed that musical training in high school music classes can induce these changes (Tierney et al., 2013), which appear to maintain during lifespan (Zendel and Alain, 2012, 2013).

**FIGURE 1:**
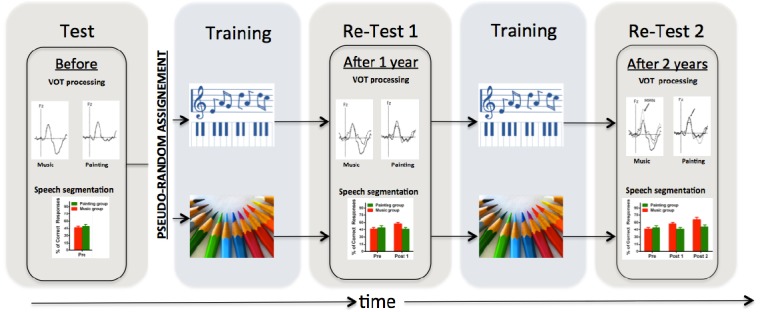
**Illustration of the experimental design used in Chobert et al. (2014) and François et al. (2013).** Using a similar design over 2 school years with test-training-retest-training-retest procedure over 2 years, 8-year-old children who followed a musical training program exhibited behavioral and electrophysiological evidence of increasing VOT processing and speech segmentation skills than children who followed a painting training program. Note that, (i) the pseudo-random assignment of the participants is crucial to control for possible confounds including, socio-economic, educational, cognitive, and linguistic measures; (ii) that the two training programs must be equally motivating, engaging, diverse; and that (iii) the training programs were provided in collective groups and not in individual.

If musicians are better in perceiving speech in noise they also have refined representations of syllables (Degé and Schwarzer, 2011; Zuk et al., 2013; see also Moreno et al., 2009) at both subcortical and cortical levels (Chobert et al., 2011, 2014; Strait et al., 2012; see Figure 1 for an illustration of the experimental design). All together, these results show that musicians have better neural encoding of speech sounds, which might help to develop a greater sensitivity to the metrical structure of speech (Port, 2003). Compared to non-musicians, adult and child musicians showed increased sensitivity to subtle pitch modifications inserted in the prosodic contour of sentences being uttered in their native or in a foreign language (Schön et al., 2004; Magne et al., 2006; Marques et al., 2007). Adult musicians are also more sensitive to the metrical structures of speech and to anomalous durational modifications in sentences (Marie et al., 2011).

Further evidence of better speech processing skills in musicians than in non-musicians was also provided by cross-sectional and longitudinal studies exploring speech segmentation ability in adults and children (François and Schön, 2011; François et al., 2013, 2014). Speech segmentation is one of the mandatory steps for acquiring a new language, which requires the ability to extract words from continuous speech. When presented with 2 min of an artificial stream of statistically structured syllables, infants and adults are able to segment and discriminate syllables sequences that are part of the stream (i.e., familiar sequences) from new sequences (i.e., unfamiliar sequences; Saffran et al., 1996; Aslin et al., 1998). While adult musicians barely outperformed non-musicians (François and Schön, 2011), musically trained children largely outperformed their non-musician counterparts after 1 year and even more after 2 years of musical training (François et al., 2013). Moreover, neural responses in both adult and child musicians differentiated familiar from unfamiliar sequences during the behavioral test. In a further study, François et al. (2014) provided evidence that the ability to differentiate familiar from unfamiliar items was correlated with how fast a fronto-central negative ERP component emerged during the exposition to the artificial speech stream.

The mounting evidence of the beneficial effects of musical training on speech auditory processing in typically developing children as well as the timing deficits found in children with language based learning impairments led researchers to test the idea that musical training could be used as a remediation tool.

### Music Training Programs in Language Impaired Populations

Overy (2000, 2003) were the first evaluating the efficiency of playing music in children with dyslexia. Despite a small group and no clear matched-control, the results showed improved phonological awareness and spelling performance after a rhythmic training program. Along these lines, short rhythmic priming sequences have be used to enhance phonological processing in typically developing children and in prelingually deaf children (Cason and Schön, 2012; Cason et al., 2015) and to improve syntactic processing in children with language impairments (Przybylski et al., 2013). Despite a clear lack of controlled trials in children and adolescents with dyslexia (Cogo-Moreira et al., 2012), a recently published study confirms that musical training can improve reading skills and educational achievement in those children (Cogo-Moreira et al., 2013). Moreover, a recent study from Bishop-Liebler et al. (2014) provides clear evidence of the positive influence of musical practice on the timing deficits found in dyslexics.

Finally, recent data suggest that an adapted musical training program can enhance auditory, phonological, and cognitive processes in 8-year-old deaf children (Rochette et al., 2014) and that musical rhythmic priming can enhance phonological production in prelingually deaf children (Cason et al., 2015). These results are important for future studies in cochlear-implanted users who show profound deficits in speech in noise perception, in the perception of complex rhythms, timbres, and melodies with somewhat preserved tempo and simple rhythms perception (McDermott, 2004; Drennan and Rubinstein, 2008).

## Neuro-Rehabilitation: Music-Based Therapies in Neurologic Population

Music playing requires the processing of multimodal information and entails high neural demands. Multimodality is an excellent opportunity to adapt different musical activities such as playing an instrument, moving in synchrony along a rhythm or listening to music with a therapeutic purpose (Pantev and Herholz, 2011). In this section, we present rehabilitative techniques using music as a key feature to remediate motor deficits in neurological conditions.

### Playing Music to Overcome Motor Deficits in Stroke

Motor impairment after stroke refers to a weakness of the muscles mainly affecting the control and performance of voluntary movements (Mohr et al., 2011). This deficit is the most common outcome after stroke with paresis of the upper and lower extremities being found in almost 70% of cases (Rathore et al., 2002). While paresis of the legs impedes functional mobility such as walking, the limitations of arm and hand paresis extend to several daily-living activities (Langhorne et al., 2011).

Music-supported therapy (MST) aims to restore paresis of the upper limb through musical instrument playing (Schneider et al., 2007; see Table 1). In order to enhance fine and gross movements, patients are trained on playing melodies on a midi piano and/or electronic drum pads. MST relies on four basic principles: massive repetition, audio–motor coupling, shaping, and emotion-motivation effects (Rodríguez-Fornells et al., 2012). Firstly, MST requires massive repetition of simple sequences of movements through the intervention. Importantly, high-intensity practice is a basic and well-accepted principle in neuro-rehabilitation (Langhorne et al., 2009, 2011). Secondly, multimodal integration may enhance audio–motor coupling where the musical sound serves as a feedback to reinforce the movement, to correct the errors, to adjust the timing and to refine motor representations. Thirdly, the therapy is adapted to the level of impairment and to the progression of the patient. Fourthly, the emotional-motivational aspects of music may regulate emotional responses through the playfulness of learning a new skill.

**TABLE 1:** **Summary of the studies evaluating MST to restore upper limb paresis in stroke patients**.

Study	Participants	MST program	Results
Schneider et al. (2007)	Subacute patients MST group (*n* = 20) CT group (*n* = 20)	15 sessions of 30 min during 3 weeks Piano and drum playing	MST group: increased frequency, velocity, and smoothness in a finger and a hand-tapping task. Improvements in ARAT, BBT, 9HPT, APS motor test CT group: no improvements
Altenmüller et al. (2009)	Subacute patients MST group (*n* = 32) CT group (*n* = 30)	15 sessions of 30 min during 3 weeks Piano and drum playing	MST group: increased frequency, velocity, and smoothness in a finger and a hand-tapping task. Increased smoothness in prono-supination movements and velocity in reaching a target. Better scores in ARAT, BBT, 9HPT, and APS motor test CT group: no improvements
Rojo et al. (2011)	Chronic patient Case study	20 sessions of 30 min during 4 weeks Piano and drum playing	Increased smoothness in a finger and a hand-tapping task and in prono-supination movements. Increased frequency in a hand-tapping task. Increased amplitude of motor-evoked potentials in both hemispheres. Reduced neural activation in the unaffected hemisphere during a motor task with the paretic hand. Functional activation of motor regions during the passive listening of trained sequences
Amengual et al. (2013)	Chronic patients MST group (*n* = 20) Healthy group (*n* = 20)	20 sessions of 30 min during 4 weeks Piano and drum playing	Increased frequency in a finger-tapping task, increased smoothness in a hand-tapping task. Better scores in ARAT motor test. A lateral shift in the representational motor cortical map. Increased amplitude of motor-evoked potentials in the affected hemisphere Healthy group: no improvements
Grau-Sánchez et al. (2013)	Subacute patients MST group (*n* = 9) Healthy group (*n* = 9)	20 sessions of 30 min during 4 weeks Piano and drum playing	Improvements in ARAT, BBT, and APS. Increased quality of life. Increased excitability in the affected hemisphere and a posterior shift in the representational motor cortical map Healthy group: reduction in the area of the representational motor cortical map
Van Vugt et al. (2014)	Subacute patients MST in turn group (*n* = 14) MST together group (*n* = 14)	Three individual sessions and seven sessions in pairs, where one group played in turns with their couple and the other group played in synchrony with their couple. In total, 10 sessions of 30 min over the course of 3 or 4 weeks Piano playing	Both groups improved in 9HPT test, but the in turn group improved more. More synchrony in a index-to-thumb tapping in both groups. Reduction in depression and fatigue in both groups. Both improved mood but the in-turn group became more positive over the therapy. The in-turn group rated higher how they experienced sessions and how they felt with partner
Villeneuve et al. (2014)	Chronic patients MST group (*n* = 13) No control group but intrasubject design	Nine individual sessions of 1 h guided by a therapist and six sessions of 30 min at home without therapist. In total, 15 sessions 3 weeks Piano playing	Better scores in BBT, 9HPT, FTN, FTT, and Jebsen motor test. Improvements mantained after 3 weeks of finishing the treatment

The abbreviations in the participants column correspond to: MST, music-supported therapy; CT, conventional treatment. The abbreviations in the results' column refers to the following motor tests: ARAT, Action Research Arm Test (Carroll, 1965;Lyle, 1981); BBT, Box and Blocks Test (Mathiowetz et al., 1985); 9HPT, 9 Hole Pegboard Test (Parker et al., 1986); APS, arm paresis score (Wade et al., 1983); FTN, Finger To Nose Test; FTT, Finger Tapping Test; Jebsen, Jebsen Hand Function Test (Jebsen et al., 1969).

Music-supported therapy is successful in reducing the motor deficits in subacute stroke patients. Altenmüller et al. (2009) and Schneider et al. (2007) compared the effectiveness of MST to conventional treatment. Only patients in the MST group improved in frequency, velocity and smoothness of fingers and hand tapping movements. Moreover, those patients obtained greater scores over time on standardized clinical tests assessing motor functions. However, to what extent is the presence of music responsible for the observed gains and associated plasticity? In a single-case study (Rojo et al., 2011), a patient performed a passive listening task with unfamiliar and trained melodies. Interestingly, while before the application of MST the patient exhibited only activation of the AC, the motor regions were also activated after the training. This phenomenon of audio–motor coupling provided evidence that the auditory feedback is an essential part of the therapy by contributing to enhance activations over motor regions (Rodríguez-Fornells et al., 2012). In a further study, Amengual et al. (2013) used TMS to demonstrate changes in the excitability of the sensorimotor cortex due to MST. Participants recovered from their motor deficits and exhibited an increased excitability of the sensorimotor cortex in the affected hemisphere after 4 weeks of MST. Moreover, a lateral shift in the motor map of chronic patients was evidenced after the training and was associated with motor gains. Interestingly, similar results have been recently reported in subacute patients (Grau-Sánchez et al., 2013). Taken together these studies suggest that MST can induce functional changes associated to brain reorganization processes.

Recent studies aimed at modifying different aspects of the MST protocol. Van Vugt et al. (2014) implemented MST in two groups of subacute stroke patients in which participants received the therapy in pairs instead of individual sessions. One group had to play together while the other group played in turns. Although both groups improved their performance in a motor task, results indicated a positive trend favoring the in-turn group. Besides, the in-turn group improved more their mood as well as their feelings about their partner. The idea that music playing is a shared experience (Overy, 2012) is interesting to consider because patients can feel emphatic and understood by others individuals presenting similar difficulties. This could in turn enhance the mood and reduce depressive symptoms (Gillen, 2010). MST has also been evaluated in a home-training protocol (Villeneuve et al., 2014) showing improvements in the paresis of chronic patients that were maintained over time. During nine 1-h sessions, patients played musical sequences using Synthesia (Synthesia LLC) a software that provides visual cues to guide them. The sessions were complemented with at home exercises on a roll-up piano. Home sessions in chronic stages may be an appropriate cost-effective approach once patients have gained a certain degree of improvements and stabilization. In this vein, the use of new technologies and adapted software with rehabilitative purposes opens new directions in the field of neuro-rehabilitation. The recently developed MusicGlove (Friedman et al., 2014) may be an alternative tool to treat paresis in chronic stages. The MusicGlove is an instrumented glove that produces notes during gripping movements while being guided with visual stimuli displayed on a screen. An exploratory study with 12 chronic patients has revealed improvements in motor functions compared to conventional therapy or isometric training (Friedman et al., 2014).

Importantly, all subacute patients involved in theses studies receive a rehabilitation program in a hospital or outpatient setting that includes physiotherapy and occupational therapy to train the affected extremity (Gillen, 2010). This may limit the interpretation of the positive effects of MST over conventional treatments because participants cannot be excluded from the standard rehabilitation program (Oremus et al., 2012). Moreover, natural brain processes of recovery take place in acute and subacute stages which may be a confounding factor (Cramer et al., 2011; Johansson, 2011; Zeiler and Krakauer, 2013). In order to control for spontaneous recovery, it is important to have comparable groups in terms of age, severity of the deficits and time since stroke. Chronic stages are characterized by a stabilization of the deficits via compensatory mechanisms at both the behavioral and neural levels (Cramer et al., 2011; Langhorne et al., 2011) and thus, it might be more appropriate to perform proper randomized controlled trials (RCT) using within-participant designs combined together with disease progression models.

### Using Music Listening to Improve Gait in Parkinson’s Disease and Other Neurological Diseases

The main symptom in Parkinson’s disease is motor impairment in gait, which is characterized by a decreased speed, shorter stride length, and asymmetries in stride times for both lower limbs, all in turn increasing cadence of steps. This pronounced reduction in speed and amplitude is accompanied by a difficulty in initiating voluntary movements in later stages of the disease, with patients experiencing a freezing of the movements (Okuma and Yanagisawa, 2008). This limitation in walking will in turn impair balance and postural control and lead patients to a reduced activity and high risk of falls (Kim et al., 2013). A dysfunction of the basal ganglia is responsible for the mentioned symptoms (Stoessl et al., 2014) and the efficacy of pharmacological treatment reduces with time. Thus, the development of behavioral therapies may help in coping with the impairment and may be an alternative to be combined with pharmacological therapy.

One approach to enhance intrinsically rhythmical movements consists in using external sensory cues to entrain the movements (Thaut and Abiru, 2010; Thaut et al., 2014). Rhythm auditory stimulation (RAS, Thaut et al., 1996) aims to facilitate gait using metronome beats. The patient is first trained to move to the beat and then the tempo is increased from 5 to 10% over the baseline to accomplish faster movements. A first study observed that 3 weeks of RAS could improve gait velocity, stride length, and step cadence more than no treatment or self-paced training program (Thaut et al., 1996). Further studies have confirmed the positive effects of RAS on gait parameters such as overcoming freezing of gate (McIntosh et al., 1997, 1998; Thaut et al., 1997; Freedland et al., 2002; Fernandez del Olmo and Cudeiro, 2003, 2005; Arias and Cudeiro, 2010; for a review, see Nombela et al., 2013). It also improves stride length, gait velocity, cadence, and asymmetry in patients with stroke (Thaut et al., 1997, 2007; for a review in other neurological conditions, see Wittner et al., 2013). The neuroplastic mechanisms beyond the effectiveness of RAS may be due to an increased activity in the cerebellum, trying to compensate the dysfunctional pathway of the basal ganglia to regions of the premotor cortex (Fernandez del Olmo and Cudeiro, 2003). However, two studies have showed more benefits in the advanced than in the early stages of the disease suggesting that the effectiveness of RAS depends on the stage of the disease (Willems et al., 2006; Arias and Cudeiro, 2008). Moreover, some studies have explored variations in RAS, manipulating the tempo (Fernandez del Olmo and Cudeiro, 2003, 2005) as well as the rhythm with respect to the individual’s baseline (Willems et al., 2006; Ledger et al., 2008). These studies have evidenced that beats presented at 20% slower than the baseline cadence does not benefit gait (Willems et al., 2006). Although other types of therapy have examined the use of other sensorial modalities (visual and proprioceptive cues), the auditory modality seems to be the best to improve gait in Parkinson’s disease (Nombela et al., 2013).

### Music Therapy and Emotion in Neuro-Rehabilitation

Beyond motor impairment, neurological patients are at high risk for suffering psychological consequences. Around one third of stroke patients suffer from depression in the following months and years (Hackett et al., 2005; Ayerbe et al., 2013) and apathy and anxiety can also be found as a frequent neuropsychiatric consequence (Campbell Burton et al., 2011; Caeiro et al., 2013). These neuropsychiatric symptoms are thought to impact health-related quality of life, increase morbidity and worsen the cognitive impairments (Whyte and Mulsant, 2002; Aarsland et al., 2012; Ayerbe et al., 2013). Psychological factors can also have a negative effect on recovery and can affect the engagement in the rehabilitation program. Pharmacological interventions have small effects on treating depression and reducing its symptoms in stroke and Parkinson’s disease and can also lead to negative side effects (Hackett et al., 2008, 2010; Aarsland et al., 2012). Some studies have reported that MST can reduce depression and fatigue and can improve the quality of life in stroke patients (Grau-Sánchez et al., 2013; Van Vugt et al., 2014). Music playing could be an alternative approach to target depression and neuropsychiatric symptoms through emotion regulation. However, there is little research studying the effectiveness of music therapy in the emotional domain. Compared to listening to audio books or auditory intervention, the daily listening to self-selected music during 2 months improves mood in the following months. Listening to music can also improve verbal memory and focused attention (Särkämö et al., 2008). Importantly, listening to music also induced structural changes with an increase of GM in frontal and limbic structures (Särkämö et al., 2014). Participants reported that music was helpful for relaxing and sleeping, influenced their mood and evoked memories and reflexive thoughts (Forsblom et al., 2009). Musical activities may be a tool to modulate the emotional reactions and cope with them through playfulness activity.

### Future Research in the Field of Music Therapy in Neuro-Rehabilitation

We focused our review on MST, RAS, and listening to music as standardized therapies to treat motor deficits and improve mood. Further research should aim to standardize interventions to build a strong dataset in this field. Moreover, the majority of studies refer to conventional treatment as the current rehabilitation program provided by the hospital. However, the content of the rehabilitation program may vary depending upon the facilities or the countries. Thus, an accurate description of the exercises performed by the control group is needed. Randomization of participants and blind evaluations of the treatment constitute RCTs and are necessary to implement interventional programs in clinical practice.

## Musical Training for Neuro-Education and Neuro-Rehabilitation: Differences and Similarities in Conceptual and Practical Aspects

Fundamental differences may exist between musical training for education or rehabilitation. Firstly, conceptual differences on the aim of musical training remain in the two cases. In neuro-education, musical training generally aims to boost a “typical” developmental trajectory (Kraus and Chandrasekaran, 2010), whereas in neuro-rehabilitation, musical training is rather used to normalize or compensate sensory, motor or cognitive deficits induced by a pathological condition (Cramer et al., 2011). Nonetheless, in the case of children with dyslexia, musical training will also aim to normalize the learning trajectories in order to facilitate speech and language processing which in turn may have a positive impact on educational achievement (Tierney and Kraus, 2013).

Secondly, the neuro-plastic mechanisms induced by musical training during childhood or in neurological population may be different. This might be due to the intrinsic physiological differences at play in these two cases. Animal and human studies showed that auditory stimulation received early in life enhances both sub-cortical and cortical electrophysiological responses to sounds (Sanes and Constantine-Paton, 1985; Kraus and Chandrasekaran, 2010) and persist in adult development (Zhang et al., 2001; Skoe and Kraus, 2012; White-Schwoch et al., 2013). Early in life, the neural system is immature and capitalizes on plastic changes such as myelination, neurogenesis, or dendritic growth (Kolb and Telskey, 2012; Kolb et al., 2012). On the contrary, stroke generally occurs in a mature neural system in which the insult has induced the death of neurons from a specific region and the disruption of neural networks. The plastic mechanisms underpinning recovery after stroke rely on different processes than in typical development such as restitution and substitution (Albert and Kesselring, 2012). Studies exploring the benefits of MST have shown that musical training induced functional reorganization of cortical motor maps (Amengual et al., 2013). In Parkinson disease, the aim of the therapy is to compensate the deficits in internally generated movements using music as an external cue that engages a different motor pathway to achieve the same goal, which is the initiation and normalization of gait (Nombela et al., 2013).

Thirdly, qualitative and quantitative practical differences in the type of training administered also remain. In neuro-rehabilitation settings, the training is generally provided during a relatively short period of time together with a high intensity (Albert and Kesselring, 2012). Interestingly, the use of the affected upper extremity during sessions of conventional stroke rehabilitation is minimal (Birkenmeier et al., 2010; Krakauer et al., 2012), suggesting that rehabilitation protocols should increase the doses of practice. In this context, musical training protocols for motor rehabilitation may be a good choice as they involve the massive repetition of movements with a high-intensity. Moreover, MST and RAS are most of the time individually administered and the complexity of the tasks is adapted to the progression of each patient. In the case of MST and particularly in middle to low-income countries, the training may become rather expensive due to the individualized administration of the therapy. However and importantly, when patients are discharged from the rehabilitation unit, they are most of the time physically inactive, exhibiting sedentary behaviors and have poor social interactions (Särkämö et al., 2008; Dontje et al., 2013; Tieges et al., 2015). The use home-based MST and RAS therapies may be important to encourage these patients to continue the rehabilitation and to maintain active. Moreover, listening to music could be good alternative not only to be applied at home but also in the rehabilitation unit, where most of the time patients remain in their rooms with no social interaction (Särkämö et al., 2008). Music making in neuro-education settings is generally provided in group settings with a lower intensity than in neuro-rehabilitation but importantly, the training is administered during longer periods of time thus allowing reaching a higher degree of musical complexity than in the context of rehabilitation. The group settings used with children may be more cognitively demanding than the ones used with neurologic patients. Most of the time, children have to play in synchrony with each other and ultimately create new musical pieces whereas patients will generally listen and reproduce simple familiar musical pieces.

Despite obvious differences, fundamental similarities might relate to the emotional, sensory, motor, cognitive, and social demands of music making *per se* (Herholz and Zatorre, 2012). In both fields, musical training is selected for its unique characteristics involving complex interactions between different domains and systems. Due to its multimodal aspect, musical training represents a good activity to develop audio–motor interactions, for cognitive stimulation and mood regulation. Moreover, music making as well as music listening are generally pleasant activities that are most likely to induce motivated behaviors. This is particularly the case for patients who may be more committed toward an enjoyable activity with a specific purpose rather than to a repetitive training with different rehabilitative tools. Musical training is also able to reinforce social cohesion or bonding through repetitive inter-individual interactions (Huron, 2001). Another similarity may also reside on the fact that musical practice will induce positive side effects: by enhancing language processing for the educational side and by boosting spared functions for the rehabilitation side. Finally, the permanence in time of the benefits of music making is clearly observed and is probably the most meaningful aspect for both purposes.

## Conclusion

The neural mechanisms of music-induced plasticity are still not perfectly understood (Fukui and Toyoshima, 2008), but the evidence for a positive effect of musical practice are growing and could justify the use of music both in the context of neuro-education (Caine and Caine, 1990) and of neuro-rehabilitation (Särkämö et al., 2014). The findings showing that the age of onset of musical training influences the dynamic of training induced plastic changes (Steele et al., 2013; Groussard et al., 2014) leads to the idea that multiple sensitive periods for specific functions and specific brain networks may co-exist in typical development (Penhune, 2011). This opens interesting perspectives to study the benefit of musical training in the developing brain as well as to study its consequences on speech perception and scholar achievement. The recent findings showing that listening to music is a rewarding experience for most of the people (Mas-Herrero et al., 2014) and that simple music listening activates the rewarding dopaminergic system (Salimpoor et al., 2013) give even more support to the idea that musical practice may be the perfect tool for neuro-education and Rehabilitation by fostering plastic changes in the healthy or pathological brains. Despite these growing evidence, the educational and health systems generally seem to be refractory to the idea of developing musical training programs. We hope that both teachers and therapists will keep on believing and applying alternative methods based on musical practice.

### Conflict of Interest Statement

The authors declare that the research was conducted in the absence of any commercial or financial relationships that could be construed as a potential conflict of interest.
